# A case study: consequences of ANTI-TNFα therapy and foot ulcerations. A patient with Ankylosing Spondylitis (AS) treated with Infliximab

**DOI:** 10.1186/1757-1146-3-S1-P6

**Published:** 2010-12-20

**Authors:** Lucy Edgson, Robert Field, Sarah Westlake

**Affiliations:** 1 Bournemouth and Poole Community Health Services, Bournemouth, UK; 2 Poole General Hospital, Dorset, UK

## Introduction

The BSR Biologics Register demonstrates increased risk of serious soft tissue infections in anti-TNFα managed patients. Mobility levels may increase with successful symptom management. However patients’ feet present with a combination of foot deformity and compromised tissue viability, with associated risk of ulceration and concomitant infection.

## Case presentation

A 50 year old male with AS presented with foot ulceration and cellulitis, following Infliximab administration. Management involved multiple antibiotics for recurrent infections and weekly podiatric care. Infliximab was stopped, leading to a flare of his arthritis and deteriorating gait and posture, complicating wound management. Active inflammation contributed to impaired wound healing.

After 8 months the patient remains off therapy. Although the chronic ulcer is improving, frequent wound / foot care is required. MRI and X-rays have been used to monitor for bone and joint sepsis.

## Discussion

This case demonstrates the impact of anti–TNFα in the presence of a foot wound. Multiple complications occur from the presence of infection and drug withdrawal. Rest increases joint stiffness and pain. It can be difficult to distinguish between infection & disease related inflammation. Drug benefits make patients reluctant to report foot problems, although in this case it was patient lack of understanding.

